# Forgoing life-sustaining treatment – a comparative analysis of regulations in Japan, Korea, Taiwan, and England

**DOI:** 10.1186/s12910-020-00535-w

**Published:** 2020-10-16

**Authors:** Miho Tanaka, Satoshi Kodama, Ilhak Lee, Richard Huxtable, Yicheng Chung

**Affiliations:** 1 Japan Medical Association Research Institute, 2-28-16 Honkomagome, Bunkyo-ku, Tokyo, 113-8621 Japan; 2 Graduate School of Letters, Kyoto University, Yoshida Honmachi, Sakyo-ku, Kyoto, 606-8501 Japan; 3 College of Medicine, Yonsei University, 50 Yonsei-ro, Seodaemun-gu, Seoul, 03722 South Korea; 4 Centre for Ethics in Medicine, Population Health Sciences, Medical School, University of Bristol, Canynge Hall, 39 Whatley Road, Bristol, BS8 2PS UK; 5 Tokyo Branch of Otani University, Shin Buddhist Comprehensive Research Institute, 2-19-11 Yushima, Bunkyo-ku, Tokyo, 113-0034 Japan

**Keywords:** Forgoing (withholding and withdrawing) life-sustaining treatment, End-of-life care, Law, Guideline, Japan, Korea, Taiwan, England

## Abstract

**Background:**

Regulations on forgoing life-sustaining treatment (LST) have developed in Asian countries including Japan, Korea and Taiwan. However, other countries are relatively unaware of these due to the language barrier. This article aims to describe and compare the relevant regulatory frameworks, using the (more familiar) situation in England as a point of reference. We undertook literature reviews to ascertain the legal and regulatory positions on forgoing LST in Japan, Korea, Taiwan, and England.

**Main text:**

Findings from a literature review are first presented to describe the development of the regulatory frameworks surrounding the option of forgoing LST in each country. Based on the findings from the four countries, we suggest five ethically important points, reflection on which should help to inform the further development of regulatory frameworks concerning end-of-life care in these countries and beyond. There should be reflection on: (1) the definition of – and reasons for defining – the ‘terminal stage’ and associated criteria for making such judgements; Korea and Taiwan limit forgoing LST to patients in this stage, but there are risks associated with defining this too narrowly or broadly; (2) foregoing LST for patients who are *not* in this stage, as is allowed in Japan and England, because here too there are areas of controversy, including (in England) whether the law in this area does enough to respect the autonomy of (now) incapacitated patients; (3) whether ‘foregoing’ LST should encompass withholding *and* withdrawing treatment; this is also an ethically disputed area, particularly in the Asian countries we examine; (4) the family’s role in end-of-life decision-making, particularly as, compared with England, the three Asian countries traditionally place a greater emphasis on families and communities than on individuals; and (5) decision-making with and for those incapacitated patients who lack families, surrogate decision-makers or ADs.

**Conclusion:**

Comparison of, and reflection on, the different legal positions that obtain in Japan, Korea, Taiwan, and England should prove informative and we particularly invite reflection on five areas, in the hope the ensuing discussions will help to establish better end-of-life regulatory frameworks in these countries and elsewhere.

## Background

Recently, two critical laws on end-of-life care were enacted in Asia. One is the Act on Decisions on Life-Sustaining Treatment for Patients in Hospice and Palliative Care or at the End of Life (hereafter, LST Decision Act), which was enforced in early 2018 in Korea [1, 2]. This Act permits the withdrawal of LST from patients at the end of life. The other is the Patient Right to Autonomy Act (hereafter PRAA) enforced in early 2019 in Taiwan [3]. Much earlier, in 2000, Taiwan enacted the Hospice Palliative Care Act, which permits withdrawal of treatment from terminally ill patients at their request. The new law allows treatment withdrawal not only from terminally ill patients, but also from any patient who is in an irreversible coma, in a vegetative state, has severe dementia, or otherwise suffers from unbearable pain.

In contrast to these two countries, Japan has yet to pass a law on forgoing LST, even though the public discussion on the issue has been ongoing for over a decade. In 2007, Japan’s Ministry of Health, Labour and Welfare (hereafter, MHLW) issued the Guideline for Medical Decision-Making Process in End-of-Life (Terminal) Care (hereafter, the Process Guideline); however, it does not explicitly permit or prohibit forgoing LST. In 2012, a group of non-partisan Diet Members proposed a draft Bill for Respecting the Patient’s Decision in End-of-Life Care Act (provisional name; hereafter, the draft AD Bill), which offers immunity to physicians if they withhold or withdraw LST in accordance with the patient’s advance directive (hereafter, AD). As of 2019, the proposed draft AD Bill has yet to be submitted to the National Diet.

As portrayed above, regulatory developments on forgoing LST have progressed in these three countries to differing degrees. Unfortunately, the nature and specifics of these remain relatively unknown to other countries, partly due to the language barrier. The legislation, guidelines, and deliberation processes are typically posted only in the native language on the websites of health ministries and parliaments of these countries, so the regulatory schemes for end-of-life care are little known beyond the particular jurisdiction, even by neighbouring Asian countries.

Many Western countries formulated public policies about forgoing LST in end-of-life care prior to most Asian countries.^1^ England is one such example, along with the US and other English-speaking countries including Australia and Canada. Looking specifically to the English legal system, professional medical organisations have long published guidance on end-of-life care, including the doctors’ trade union the British Medical Association (hereafter, BMA) and the doctors’ regulator in England, the General Medical Council (hereafter, GMC)^2^^,^^3^^,^^4^^,^^5^ English law primarily derives from the common law (i.e. the decisions of judges) and Acts of Parliament. Although common law had long governed LST decisions, in 2007 the Mental Capacity Act 2005 (hereafter, MCA) came into force, which covers LST decisions for adult patients who lack “mental capacity” [4].

Comparison of the situations in Korea, Taiwan and Japan with the situation in England is useful as it enables clarification of legal and philosophical issues pertaining to the legalisation of forgoing LST. We believe that the results of such a comparison will be valuable for critical reflection on the relevant laws and policies, in these countries and others. To this end, the present study involved a literature review to examine the laws, guidelines, and newspaper articles relevant to this issue. Below, we first summarise the development of regulatory frameworks on forgoing LST in Japan, Korea, Taiwan, and England. We then compare the regulatory frameworks on forgoing LST in each country. Finally, we discuss legal and philosophical issues concerning forgoing LST that need to be addressed in order to create better regulatory frameworks for end-of-life care in Asian countries and worldwide.

## Main text

### Development of regulatory frameworks with regard to forgoing LST in Japan, Korea, Taiwan, and England

#### Japan

In Japan, court cases and other incidents highlighting problems associated with LST withdrawal increased the momentum for legislation. While no Bill has been submitted to the National Diet for deliberation, several ethical guidelines have been created. Here, in order to better understand the current situation in Japan, we summarise two crucial court cases and one incident regarding the forgoing of LST.

##### The Tokai Case (1991)

In this case, the attending physician withdrew treatment from a 58-year-old patient suffering from end-stage multiple myeloma at Tokai University Hospital. The physician then administered sedative drugs to stop the patient from stertorous breathing, followed by potassium chloride, which causes heart failure if injected undiluted. The patient subsequently died from cardiac arrest, and the attending physician was prosecuted for homicide. In March 1995, the Yokohama District Court found the attending physician guilty of murder and sentenced him to 2 years in prison with 2 years of suspension [5]. In the ruling, the Court not only set forth admissibility requirements for active euthanasia, but also provided admissibility criteria for forgoing LST in the *obiter dictum* (Table 1). Although the Tokai Case concluded at the district court level without appeal, the decision had a considerable impact on Japanese society and influenced subsequent discussions, not only on active euthanasia, but also concerning the forgoing of LST.

**Table 1:** Admissibility requirements for forgoing treatment established by the Yokohama District Court in the Tokai Case^a^

Three admissibility requirements for forgoing LST	
1. The patient has no hope of recovery and death is imminent.	
2. It is desirable that the patient has declared his or her wishes when forgoing treatment is considered an option. It is permissible to presume the family’s will to be the patient’s will, provided that the patient’s family makes a deliberate decision that takes into consideration the patient’s perspective.	
3. Treatments that can be forgone include all treatments considered to be curative measures, supportive measures, or LST, e.g., drug administration, chemotherapy, artificial dialysis, artificial ventilator, blood transfusion, and artificial nutrition and hydration (ANH).	

^a^The term they actually use in the decision is “stop treatments (chiryo chushi),” but because there appears to be no distinction in the decision between withholding treatments and withdrawing them, we interpreted the word “stop” to mean “forgoing”

##### The Kawasaki Case (1998)

In the case of Kawasaki Kyodo Hospital, the attending physician removed the endotracheal tube from a 58-year-old patient upon request from the patient’s family. The patient was unconscious due to hypoxic brain injury accompanied by status asthmaticus [6–8]. When the physician could not alleviate the patient’s heavy breathing, she ordered an assistant nurse to inject a muscle relaxant, and the patient eventually died. The doctor was later charged with homicide.

The most distinctive aspect of the Kawasaki case was that the disputed point in the Supreme Court was the illegality of treatment withdrawal. Both the Yokohama District Court and the Tokyo High Court in the Kawasaki Case decided that treatment withdrawal was illegal because two requirements for withdrawing treatment had not been fulfilled, specifically “the physician’s duty of care” and “the patient’s autonomy”, respectively corresponding to the first and second admissibility requirements in the Court decision of the Tokai Case. The physician was accordingly convicted of homicide. In early 2007, the Tokyo High Court sentenced the physician to 1 year in prison with 3 years of suspension. In 2009, the Supreme Court ruled to uphold the High Court ruling.

##### The Imizu Incident (2000-2005)

In the Imizu Municipal Hospital incident, two physicians withdrew artificial ventilators from seven terminally ill patients in their 50s to 90s in response to requests either from the patients or their families [9, 10].^6^ All patients died following removal of the ventilators. In 2006, the incident made national headlines, with the hospital director apologising that his staff had undertaken “ethically problematic” treatment withdrawal. In 2008, the police referred the physicians to public prosecutors. By the end of 2009, however, the Public Prosecutors Office decided not to prosecute due to insufficient evidence [11].

Both the Kawasaki Case and the Imizu Incident fuelled the fears of healthcare professionals about facing legal or social sanctions over forgoing treatment, and clarification of the legal (im)permissibility of forgoing LST became an urgent issue for the government and professional medical associations. In 2007, soon after the High Court Decision on the Kawasaki Case and the exposé of the Imizu Incident, the MHLW issued the Process Guideline, which addressed the issue of LST withdrawal (details explained below in Section 2) [12]. Several professional associations also published end-of-life guidelines, including the 2014 Guideline on End-of-Life Care in Acute Care and Intensive Care (compiled collaboratively by the Japanese Association for Acute Medicine, the Japanese Society of Intensive Care Medicine, and the Japanese Circulation Society) [13], the 2007 Guideline on End-of-Life Care (Japan Medical Association) [14], and the 2012 Guidelines for Decision Making Process of Elderly Care: Focusing on the Use of Artificial Hydration and Nutrition (Japan Geriatrics Society) [15].

However, even after publication of the MHLW’s Process Guideline and other professional guidelines, healthcare professionals were not sure about the legality of forgoing LST. For example, in 2008, 1 year after the publication of the MHLW’s Process Guideline, the ethics committee at the Kameda Medical Center approved the withdrawal of the artificial ventilator from a patient with Motor Neurone Disease (MND), but the hospital director chose not to follow the committee’s advice due to legal uncertainty [16–18].^7^^,^^8^^,^^9^ This situation led to the drafting of an AD Bill in 2012 by a group of non-partisan Diet Members.^10^ Seven years later, the draft AD bill has yet to be submitted to the National Diet.

#### Korea

In Korea, the Boramae Hospital Case and the Severance Hospital Case were critically important in shaping the public discussion and subsequent legislation on LST withdrawal.

##### The Boramae Hospital Case (1997)

The attending physician at Boramae Hospital removed the artificial ventilator from a 58-year-old patient, who had been admitted to an intensive-care unit (ICU) after a surgical operation due to massive intracranial haemorrhage, following a request from the patient’s wife [19, 20]. The doctor and the patient’s wife were subsequently charged with homicide and prosecuted as joint offenders. The medical team in the hospital had tried to persuade the wife to continue the patient’s treatment in the hospital, but she requested that the patient be discharged because of the financial burden of further hospitalisation, and the team decided to discharge the patient. Less than 5 min after leaving the hospital, the patient developed respiratory difficulties and died. Expecting to be the beneficiary of a free funeral service if police were engaged, the wife reported this as an unexpected death to the police, and it led to a court case.

In 2004, the Supreme Court sentenced the physician to 18 months in prison and 2 years of probation. The Court ruled that if the physicians discharged the patient at the request of the patient’s wife and were aware that the reason for the wife’s discharge request was the death of the patient and that discharge of the patient would lead to his death, then the physicians were guilty as accomplices to murder. This ruling came as a shock to many Korean physicians. Until then, proxy decisions by the patient’s family had been generally accepted as essential grounds on which physicians could make difficult choices regarding treatment. However, the court decision reminded physicians of their duty to act in the best interests of the patient before complying with the will of a (potentially untrustworthy) proxy. However, physicians regarded this decision as requiring them to do their best to prevent the death of the patient [21].

##### The Severance Hospital Case (2008)

A 77-year-old female patient at Severance Hospital suffered hypoxic brain damage due to severe bleeding from the pulmonary artery, which was a complication arising during bronchoscopic biopsy [20]. She was deemed to be in a persistent vegetative state (PVS) and could not breathe sufficiently on her own. Her family asked the hospital to withdraw the artificial ventilator from the patient, but the hospital refused. The family filed a lawsuit and won the case at the Supreme Court level.

This case was different from the Boramae Hospital Case in two respects. First, the patient was thought to be irreversibly dying.^11^ Second, the Court judged the family’s proxy decision to be the patient’s will. Furthermore, the Supreme Court pointed out the necessity of legislation on forgoing LST that respects the right to pursue happiness, as guaranteed by the Korean Constitution.

Along with these court cases, the following significant events happened in Korean society [19, 20]. In 2002, in response to the Boramae trial, the Korean Association of Medical Societies (KAMS) proposed the KAMS Guideline on Forgoing LST. In 2009, in response to the Severance case, the KAMS, the Korean Medical Association, and the Korean Hospital Association together established a set of guidelines on forgoing LST. Creation of these guidelines attracted the attention of legal and ethical professionals, policymakers, and medical professionals [20]. Several Bills on withdrawing LST were submitted to the National Diet from 2006 to 2015. These developments led the government to set up consultation bodies in 2009 and to take measures to facilitate social consensus-building around these topics. The topics included whether or not treatments may be withdrawn only from terminally ill patients, whether LST (including cardiopulmonary resuscitation and artificial ventilation but not ANH) may be withdrawn, and whether and to what extent physicians must respect patient autonomy. These developments culminated in the enactment in 2016, and full enforcement in 2018, of the Hospice Palliative Care and LST Decision Act.

#### Taiwan

Taiwan was one of the first Asian countries to create a law on forgoing LST.^12^^,^^13^^,^^14^ It is believed that the practice of “terminal discharge from hospital” played an important role in shaping the public discussion and subsequent legislation concerning LST. In Taiwan, the term “terminal discharge” refers to the practice of allowing terminally ill patients to spend the last few hours or days with their families at home by being discharged from the hospital of their own will or their family’s proxy decision. The legitimacy of such practice was recognised as part of the “discharge at patient’s request,” which was stipulated in the Medical Care Act in 1986 [22, 23]. Since then the practice of “terminal discharge” has been widely accepted in the clinical scene in Taiwan.

Along with the above development, the hospice movement began to take root in Taiwan [22, 24]. In 1983, a substantial discussion on the need for hospices and palliative care began, and the first hospice program was set up in 1987, followed by the creation of the first hospice unit in Taiwan in 1990 [25]. Since then, several hospice wards have been established in both public and private hospitals, and many religious hospice foundations and non-profit hospice organisations have been created.

As the hospice movement developed in Taiwan, calls for the legalisation of forgoing LST grew amongst healthcare professionals in 1989. The Ministry of Health and Welfare (formerly the Department of Health; upgraded as MHW in 2013) was initially reluctant to answer such requests because the officials believed it was too early to discuss such an issue. However, as the hospice movement progressed in Taiwan, the MHW indicated in 1996 that providing palliative care and withholding LST were within the realm of a doctor’s “duty of care” and, therefore, not illegal. Following the policy change in MHW, healthcare professionals sought legislation on Do Not Resuscitate (DNR) orders, in order to allow patients to die naturally in hospital [22]. As a result, the Hospice and Palliative Care Act was enacted in 2000, legalising the withholding of LST from terminally ill patients in line with their ADs (including a healthcare proxy to legally make healthcare decisions on behalf of patients) or their family’s consent. The law was amended several times and now also permits LST withdrawal at the patient’s or family’s request, as described in more detail in Section 2 below.

Although the Act contributes to the development of Hospice in Taiwan, it was criticised for not fully respecting patient self-determination for two reasons. First, the physician’s duty to tell the truth, to disclose a diagnosis, or to obtain consent from the patient is not explicitly stipulated [26]. Second, even after the law was enforced, the patient’s family primarily make decisions to withhold or withdraw LST [27]. In response to these criticisms, the PRAA was passed at the end of 2015 and enforced in January 2019.

#### England

The law and professional guidance permit LST to be withdrawn or withheld, not only from patients who have mental capacity and have refused such treatment but also from patients who lack mental capacity. The relevant principles were initially enshrined in professional guidance and court rulings, but some have since been set down in the MCA. The crucial first ruling in this area was *Bland*, which concerned a patient who lacked the mental capacity to make decisions about his treatment at the relevant time [28, 29].

##### The Bland Case (1989)

Anthony Bland (then 18-years-old) was trapped in a crush at the Hillsborough Football Stadium disaster in Sheffield (England) and later diagnosed to be in a PVS. Three years passed without any improvement in his condition. He had not indicated his will concerning the treatment in advance. However, the hospital Trust and Bland’s family came to believe that treatment withdrawal was appropriate as they perceived no benefit in treatment continuation. The hospital applied to the court, seeking a declaration that the withdrawal of Bland’s ANH would be lawful.

In 1993, the House of Lords, the then superior court, ruled that withdrawing LST is an omission, which can be justified if a responsible body of doctors hold that treatment is not in the patient’s best interests [30, 31]. The court ruled that future such cases involving patients in the PVS should be brought before a judge for a decision as to whether or not LST should (continue to) be provided. After the decision, Bland’s ANH was withdrawn, and he died shortly afterwards.

PVS cases involving ANH were, therefore, a matter for the judges and the requirement was subsequently extended to patients in the minimally conscious state (MCS) [32]. However, the (now) superior court, the Supreme Court, has recently confirmed that there is no requirement to bring such cases to court [33]. This means that, regardless of the patient’s diagnosis or the treatment to be withdrawn, decisions do not require court approval, although difficult or contested situations may still come before the judges. Instead, LST decisions are generally a matter for the healthcare professionals, albeit in consultation with the incapacitated patient’s loved ones. Following the Bland case, professional societies issued guidelines on forgoing LST. These included the BMA, which published guidance on withholding and withdrawing LST in 1999 [34]. The GMC also published guidance, entitled “Withholding and withdrawing - guidance for doctors” in 2002, which set the standards of practice expected of doctors when they consider whether to withdraw or withhold treatments that might prolong a patient’s life [35]. In 2010, the latter was replaced with new guidance, entitled “Treatment and care towards the end of life: good practice in decision making.” The GMC’s current guidance provides a framework for good practice regarding the provision of treatment and care to patients who are likely to die within a year [36]. There are also more specific guidance documents, focused on (for example) decisions about CPR and, most recently, ANH [37, 38].

The various professional guidance encompasses not only incapacitated patients, but also patients who have “mental capacity” (or competence). The common law had long defined capacity in functional terms, i.e. the focus was on whether the patient could comprehend and retain relevant information and reach a decision [39]. The common law had also long recognised the right of a competent adult patient to refuse LST, a position most powerfully confirmed in *Re B* in 2002, in which a ventilator-dependent patient had this unwanted treatment withdrawn at her request [40]. Furthermore, the common law also recognised an adult patient’s right to make an AD refusing treatment they might otherwise receive in the future when incapacitated: provided that the patient was competent and informed, and their advance directive applied to the situation that later arose, their refusal had to be respected [41].

Many of these propositions were then enshrined in the MCA in 2005, thus replacing the common law [4, 42]. As the Parliamentary Office of Science and Technology reported, “while healthcare practice had to operate according to these common law judgements, historically it has been guided by more paternalistic concepts of duty of care without a strong legal awareness. Outside of hospitals, however, for many carers of people with learning disabilities, dementia or mental illness, concerns were expressed about the lack of legal guidance [43]”. The MCA, therefore, provided a statutory test for mental capacity and generally provided that decisions about patients who lacked capacity were to be made in their “best interests”. Although there is no strict legal test of best interests, the courts have increasingly emphasised the importance of respecting the patient’s wishes and values [44]. Furthermore, the Act also placed ADs – specifically now labelled “advance decisions to refuse treatment” – on a statutory footing, and for the first time provided for surrogate decision-making, through the creation of “lasting powers of attorney” (hereafter LPA). Details of the law are explained below.

### Comparison of regulatory frameworks in the four countries

This section compares the regulatory frameworks of the four countries regarding forgoing LST. The foci under comparison include: the legal effectiveness of advance directives; eligibility criteria for medical conditions; the definition of ‘terminal phase’; and proxy decision-making by the patient’s family.^15^

#### Japan

In Japan, the Tokyo High Court ruling in the Kawasaki Case highlighted the need for a law or guidelines on forgoing LST, yet no relevant law has been enacted to date. The MHLW’s Process Guideline issued in 2007 has been the most influential document in this area to date.^16^ It consists of the main body and commentary, and contains two core elements: 1) respecting patient self-determination and 2) deciding the course of care by the healthcare team (and not by the attending physician alone). The Process Guideline stipulates that the healthcare team shall make healthcare decisions through repeated discussions with patients and their families, with a particular emphasis on respecting the patient’s will. It also specifies that if patients cannot express their will, the healthcare team shall decide the best course of care in light of the family’s wishes. The Guideline further states that if it is difficult for the healthcare team to decide or reach a consensus with patients or their family, a committee composed of multiple experts should be set up for consultation. In this way, the Process Guideline puts a significant emphasis on consensus-building among those involved in the patient’s end-of-life care.

Interestingly, the Process Guideline is silent on the following two points. First, the main body of the Process Guideline does not define “the terminal stage” or when the patient is considered as terminal or facing the end-of-life stage, even though this phrase appears in the very title of the Guideline [45].^17^ The commentary of the Guideline does state that there can be different types of terminal stages according to the patient’s disease, which could include terminal cancer, chronic disease, cerebrovascular disease, and senility. The healthcare team is instructed to judge when the patient has reached the terminal stage through careful assessment of the patient’s condition. However, the Guideline does not provide any specific criteria that would define the terminal stage, such as “six months to live”. Second, it is unclear whether healthcare professionals can avoid criminal or civil liability for forgoing LST if they act according to the Guideline. After all, the Process Guideline is not legally binding because, although the MHLW issues it, it is not supported by any specific law.

#### Korea

In Korea, the LST Decision Act was passed in January 2016, and full enforcement of the law began in February 2018. As the name of the Act implies, it allows patients with not only cancer but also other medical conditions to receive hospice and palliative care. Additionally, it enables people to create ADs about withdrawing LST and sets up a national online registry system for storing ADs and reviewing them.^18^ Physicians are granted access to the registry and are therefore able to access an AD, which can inform decision-making. The Act also stipulates that a consensus between two or more family members can be regarded as the patient’s will when the patient does not have an AD or cannot express his or her wishes.

Characteristically, the Korean act gives a two-fold definition of “end-of-life”. “Terminal stage”, which is used to define the terminal patient, is when a patient is expected to die in several months, and the provision of hospice palliative care is considered appropriate. “The end of life process” refers to situations where a patient’s death is considered imminent due to rapid deterioration of his or her condition and the withdrawal of LST is offered as a choice. While clarifying the necessity of hospice care, the Korean law is restricted in its application to LST decisions, because an LST decision is only applicable at the very end of a disease process.

The Act also provides a clause covering a physician’s order on LST (Life-Sustaining Treatment Plans), which is a physician’s order written after consulting a patient about her wishes about end-of-life care. The physician may (or the patient may ask the physician to) inform the patient about diagnosis, end-of-life care options, and LST options, and prepare this document. When a physician places this document in the healthcare record, it takes priority over any existing AD that purports to state the patient’s preferences on LST. In a sense, this order resembles the Physician Order for Life-Sustaining Treatment (POLST) used in the various American States. However, the Korean document is supposed to be used within hospitals, whereas POLST is focused on emergency medical services.

#### Taiwan

As explained above, Taiwan has two laws concerning forgoing LST. The Hospice and Palliative Care Act allows withdrawing and withholding LST from a terminally ill patient who has a valid AD, which covers the relevant treatment options and designates healthcare proxies; if there is no AD, the patient’s family can submit a consent form on their behalf when patients cannot express their will. The phrase “terminally ill patient” refers to a person who is diagnosed by a physician as having an incurable disease or injury and whose death is imminent based on the best available medical evidence.

The PRAA, which was enacted in January 2016 and enforced in January 2019, explicitly stipulated that healthcare institutions and physicians are duty-bound to tell patients about their medical conditions, treatments, procedures, medications, and prognoses in order to honour the patient’s right to know. It also stipulated that, with a valid AD, LST (including ANH) can be withdrawn from not only terminally ill patients but also patients in an irreversible coma, a persistent vegetative state, and from those with severe dementia or other medical conditions specified by the ordinance of the MHW [3].

Although both laws stipulate forgoing LST, the PRAA is complements the Hospice and Palliative Care Act by focusing on a patient’s autonomy. As the PRAA expands the clinical conditions to non-terminally ill patients and includes ANH in the range of LST, advance care planning (ACP) must be conducted before any AD is made. Such legal regulation of ACP is new to Taiwanese society because, according to the Hospice and Palliative Care Act, consensus-building among the involved parties is not a necessary condition to forgo LST. The decision of forgoing LST for terminally ill patients can be made either with a patient’s AD or with their families’ consent. Since Taiwan has little experience of ACP, this has been a major focus of work since the enactment of PRAA in 2015: hospital staff are being trained in this area and ACP groups, which consist of a physician, a nurse, and a social worker, are being developed. After the enactment of PRAA in 2016, the development of ACP groups in hospital and provision of suitably knowledgeable hospital personnel has been the main tasks of the enforcement in 2019 [46].

#### England

In England, there are many legal developments and professional guidance documents pertinent to the issue of forgoing LST, but for the sake of brevity, we focus on the critical legislation and professional guidance. First, the MCA focuses on decisions made about, with and for adults who lack mental capacity. Like the preceding common law, the Act essentially defines capacity in functional terms [47]. If a patient has capacity, they may prepare an advance decision to refuse treatment that might otherwise be provided when she or he lacks capacity. Such a decision can apply to LST, provided that the decision is written, signed and witnessed, and there is a clear statement to the effect that the decision will apply even if this will put the patient’s life at risk [48]. The MCA provides immunity for physicians who withhold or withdraw treatments from their patients if they reasonably believe that an advance decision exists that is valid and applicable to the treatment in question [4]. However, the Act does not require the patient’s medical condition to be terminal in order to execute the patient’s AD to forgo treatments.

The MCA also provides for surrogate decision-making, through the LPA. The Act also empowers the court to appoint a deputy to make decisions, for example, in cases where there is a serious and enduring lack of capacity, such as due to dementia. However, the LPA is the main means by which a patient can appoint someone to decide on his or her behalf. The MCA stipulates that an attorney has the power to consent to or refuse LST if the patient has explicitly so empowered the attorney. Notably, however, an LPA is required to make decisions in the best interests of the patient; as such, there is always the possibility that an attorney’s decision will be challenged on this basis.

Indeed, for patients who lack an AD or LPA, best interests provide the test as to whether treatment – including LST – should be provided. The Act enumerates, rather than elaborates, the factors to be balanced in a best interests decision [49]. The law in this area had evolved considerably since *Bland*, with the courts increasingly emphasising the holistic nature of the assessment, i.e. medical factors, which had been dominant in the 1993 ruling, were not the only consideration [50]. The MCA seeks to emphasise the breadth of the test, as have the courts – including the Supreme Court, the now superior English court – in subsequent rulings [51]. Clinicians are required to consult appropriately in determining a patient’s best interests. If there is no one close to a patient to provide input into the decision, the Act also provides an Independent Mental Capacity Advocate (hereafter IMCA) service, which can support decision-making by and for incapacitated patients in their best interests [52, 53].

A further recent legal change concerns patients with prolonged disorders of consciousness. *Bland* had required decisions about ANH for patients in the PVS to come to court, a requirement which was later extended to patients in the MCS. Although that requirement persisted for decades, recent rulings have confirmed that this is not strictly the law: as such, these cases should no longer routinely come to court, although they may do so, for example, if there is disagreement between the relevant parties about what is in the patient’s best interests [33].

The second crucial regulatory source is the GMC guidance on decision-making in end-of-life care. The GMC guidance addresses issues surrounding decision-making in end-of-life care and provides decision-making models for patients who can decide as well as those who lack the capacity to do so [54].^19^ The guideline advises physicians to provide or forgo LST when they judge that these would not be clinically appropriate for a patient. When making such judgments, physicians must weigh the benefits of treatment against the burdens and risks for the patient. Furthermore, physicians should carefully take into account not only clinical considerations but also other factors relevant to the circumstances of each patient, including the patient’s wishes, values, and feelings. It also emphasises the importance of ACP. Unlike the MCA, the guidance defines the phrase, “approaching the end of life”.^20^

### Legal and philosophical issues

In this last section, we will discuss the legal and philosophical issues that have emerged from the comparison of regulatory frameworks relating to forgoing LST in Japan, Korea, Taiwan, and England. In order to bring about improvements in these frameworks, particularly concerning end-of-life care, the following five points must be addressed.

### Defining ‘terminal stage’

First, the definition of terminal stage (or phase) can be essential when considering a regulatory framework for forgoing LST. As described in Section 2, the LST Decision Act in Korea and the Hospice and Palliative Care Act in Taiwan permit patients to forgo treatment only in the terminal stage. It makes the definition of ‘terminal stage’ significant, not to mention the clinical judgement of when a particular patient is in the terminal stage.

Both Korean and Taiwanese laws stipulate that a patient is in the terminal stage when his or her condition is diagnosed as irrecoverable and his or her death is imminent with rapid deterioration of the condition. As explained earlier, the Korean Act distinguishes between two phases of the terminal stage; namely, the terminal stage when patients with irrecoverable conditions are expected to die within several months, and the end-of-life stage when patients are facing imminent death with rapidly worsening conditions. The Act only allows the latter patients to elect for withdrawal of LST. Meanwhile, the Taiwan Act interprets the terminal stage more conservatively and considers terminal patients to be those who have been diagnosed as having an incurable disease or injury, and who cannot avoid death shortly based on the best available medical evidence. This definition more or less corresponds to “the end of life stage” in the Korean Act.

Regarding the kind of medical conditions to which the law applies, the Korean law limits the “terminal stage” to patients with cancer, acquired immune deficiency syndrome (AIDS), chronic obstructive pulmonary disease (COPD), and chronic hepatic disease, as well as other diseases specified by the Ordinance of the MHW.^21^ Patients with these conditions are eligible for hospice palliative care in the terminal stage, while no such limitation exists for patients in the dying stage who are eligible to elect for termination of LST. On the other hand, although there are similar rules about the coverage of palliative care in the Taiwanese National Health Insurance system, the Hospice and Palliative Care Act in Taiwan does not specify any health conditions for patients in the terminal stage to be eligible for forging LST.

In Japan, no law stipulates a definition of terminal stage. The MHLW’s Process Guideline avoids providing a definition and only suggests several instances of medical conditions which can be considered as reaching a terminal stage, such as “when a patient’s prognosis is predicted to be from a few days to 2-3 months in the case of terminal cancer”.^22^ England also lacks such a stipulation; indeed, LST may be refused, withdrawn or withheld regardless of the patient’s life-expectancy. The MCA, for example, is not limited to patients in the terminal stage [4]. The GMC guideline, however, states that patients are “approaching the end of life” when they are likely to die within the next 12 months, which is broader than the Korean or Taiwanese criteria.

We believe that the definition of terminal stage should be neither too narrow nor too wide when specifying the period within which one can choose to withdraw treatment. If the definition is too narrow and the law only applies to patients who are expected to die within a few days or even a few hours, the case for withdrawing LST might not be so compelling, for they will die in a short time in any case. That said, however, there may be clinical (and ethical) reasons for withdrawing treatment, even in such a short period, to ensure that the imposition of treatment does not adversely affect the dying process, and there may also be cultural reasons for doing so, as demonstrated by Taiwan’s “terminal discharge from hospital”. On the other hand, if the definition is too broad, the law might apply to those with incurable diseases such as MND, end-stage renal disease (ESRD), and Alzheimer’s disease, who may be able to live for a relatively long period if treatment – such as artificial ventilation, dialysis, or gastric fistula – is continued. However, expanding the definition to include patients with such conditions might give rise to concerns and confusion, particularly as such a definition would potentially include those who are early in the disease trajectory, and therefore not in the “terminal phase” as this is conventionally understood. Moreover, patients who are diagnosed with such incurable diseases might feel threatened because the regulatory framework such as law or guidelines would classify them as “terminal patients” who can have their treatments withdrawn. Of course, the requirement that the patient must consent might dispel any concerns these patients and their loved ones might have – but we should also be mindful that such patients might feel under pressure to “consent” [55].

Alternatively, a strict definition of the terminal stage could be avoided altogether. If the principle of autonomy or the right of self-determination is considered paramount, then perhaps we may not need to limit forgoing treatment to the terminal stage, as England’s MCA and Taiwan’s new law seem to suggest. However, if we adhere to the idea that the patient can choose to have LST withdrawn only at the terminal stage, then a defined set of clinical criteria for “terminal stage” are likely to be required.

Based on our analysis, one important lesson is that the purpose of defining the terminal stage should be clear. In particular, two relevant objectives for defining the term would be the specification of a period when retreating from aggressive treatments and focusing on palliative care is appropriate, and the determination of a period when the patient or the family can choose to withdraw LST. Furthermore, it appears that policymakers and professional societies should be required not only to define the term but also to establish clinical criteria for judging when patients with various conditions are in the terminal stage.

### Forgoing LST for non-terminal patients (e.g., patients with MND and those in PVS)

The second issue is whether to allow patients who are not in the terminal stage – but are suffering from conditions such as MND or are in a PVS – to forgo LST. Such patients who are not in the terminal stage can continue living with the assistance of artificial ventilators and ANH.

Our comparison has revealed different positions on withdrawing or withholding LST from these non-terminal patients. The Korean Act, for example, limits the permissibility of treatment withdrawal to the dying stage, thereby excluding non-terminal patients with PVS or MND. Taiwan’s Hospice and Palliative Care Act also applies only to terminal patients. However, the newly enacted PRAA has a broader scope than the preceding Act and allows even PVS patients to forgo LST. It should be emphasised here that MND is not included in the four conditions stipulated in Taiwan’s new Act. England’s MCA does not have any such restrictions and thus applies to both terminal and non-terminal patients. In Japan, the draft AD Bill does not mention specific medical conditions that would fall under the scope of the Bill; presumably, however, neither the draft AD Bill nor the MHLW’s Process Guideline applies to non-terminal PVS or MND patients.

Not allowing MND patients to withdraw LST because they are not terminal seems particularly problematic in places like Japan, where tracheostomy with invasive mechanical ventilation (TIV) rates for MND patients may be the highest in the world [56], ranging from 24.5 to 45.9% [57–59]. Even if MND patients require artificial ventilators or ANH, they can remain alive if they choose to receive these treatments. If we understand the terminal stage as an incurable and irreversible condition that, regardless of the use or withdrawal of LST, will result in death within a relatively short time [60, 61]^23^, then these MND patients are not terminal. In Japan, however, although the MND patients’ choice of withholding LST seems to be respected [62], detaching artificial ventilators once they have been initiated is next to impossible [63] and physicians who detach them from MND patients are highly likely to be prosecuted [56, 64].^24^ In such circumstances, MND patients can choose either to receive or not to receive LST, knowing that this cannot later be withdrawn even if they desire it.

In these Asian countries, whether or not to allow PVS and MND patients who are not in the terminal stage to forgo LST remains controversial. England has also struggled with the question of whether LST can be withdrawn from MCS patients. The courts initially appeared reluctant to authorise the withdrawal of ANH from such patients, although they have become more willing to countenance this, at least where there is compelling evidence that the patient him- or herself would not consent to such treatment [65, 66]. Whether the law in this jurisdiction does enough to respect the autonomy of (now) incapacitated patients nevertheless remains a live issue [67].

### Moral and legal differences between withdrawing and withholding LST

In most Western countries including England, the common understanding is that there is no morally significant difference between withholding and withdrawing treatments [29, 68, 69]. Thus, withdrawing treatments is permitted in circumstances in which withholding treatments is permitted [70].

In contrast, such a view is not necessarily the norm in Asia. According to a 2012 survey of physicians who manage patients in intensive care units (ICUs) in 16 countries and regions in Asia, the proportion of respondents who felt that withholding and withdrawing LST were ethically dissimilar was 75% on average [71]. By country or region, the largest was 90% in Pakistan, followed by 86% in Bangladesh, while the lowest was 41% in Singapore, followed by 49% in Hong Kong. In Japan, 71% responded that withholding and withdrawing were ethically dissimilar; the percentages were 79% in Korea and 80% in Taiwan. For example, in clinical settings in Japan, the general idea is that withdrawing LST is ethically more difficult than withholding it, and that withdrawing LST is considered “unacceptable” [69, 72].

Legal scholars in Japan are also split on the legality of withdrawing LST. For instance, according to one legal scholar, “no medical professional has been prosecuted for removing the artificial ventilator or the gastrostomy feeding tube from a terminally-ill patient” since the publication of the Process Guideline by the MHLW in 2007 [73, 74], implying that withdrawing LST is not illegal if one follows the protocol set by the Process Guideline. On the other hand, some scholars think that, whereas withholding treatment can be regarded as an instance of omission, it is difficult to view withdrawing as such, and thus claim that withdrawing and withholding treatment is not necessarily legally equivalent [75, 76]. Thus, while an absolute consensus exists regarding the legal status of withholding LST, differing views are held on withdrawing LST, not only among healthcare professionals but also among legal scholars.

Several factors may underlie the difficulty of equating withholding LST with withdrawing it. For example, a qualitative survey of emergency physicians in Japan conducted between 2006 and 2007 found the following factors to be motivations for avoiding the withdrawal of artificial ventilators: 1) fear of criminal prosecution and concern about unwanted media exposure; 2) concern for the feelings of the patient’s family; 3) physicians’ psychological barriers to shortening patients’ lives by withdrawing LST because they regard withdrawing LST to be an act and not an omission; and 4) medical factors in the acute phase of a severe condition, including the uncertainty of treatment outcome (i.e., they cannot completely deny that the patient may recover) [77]. The fact that there is no law in Japan permitting the withdrawal of LST may also be a background factor.

In summary, unlike their counterparts in Western countries, many healthcare professionals, as well as some legal scholars, in Asian countries still find it difficult to equate withholding LST with withdrawing LST. Moreover, as the above discussion suggests, it may be necessary to address the feelings of healthcare professionals adequately when considering a legal framework for end-of-life care.

### Role of the family in end-of-life decision-making

Fourth, our comparative analysis revealed that the role of the family (or, indeed, other loved ones) in England differs from those in the three Asian countries. Although the family plays a significant role in end-of-life decision-making in all three Asian countries, the legal stipulations concerning their role are slightly different. In Taiwan, the Hospice and Palliative Care Act stipulates the role of the family in end-of-life decision-making. The Act states that patients may nominate a medical proxy in advance and that the designated family member by law may consent to forgo LST on behalf of terminally ill patients if their will cannot be expressed and no AD has been signed. The family members designated by law include (in descending order of closeness): spouses, adult children and adult grandchildren, parents, adult siblings, grandparents, great-grandparents or collateral consanguinities within the third degree, lineal relatives by affinity within the first degree. The law states that one designated family member should sign the consent form for forgoing LST. If there are disagreements concerning forgoing LST among family members, the consent of the closer family member should be prioritised following the order of closeness.

In Korea, the LST Decision Act defines two aspects of surrogate decision making, which involves family members verifying the patient’s intention by witnessing (Article 17) and deciding for the patient (Article 18). The Act also sets out the differing family input in each of the cases. The family members considered relevant for witnessing are: 1) spouse, 2) linear descendants, such as children and grandchildren, 3) linear ascendants, such as parents and grandparents, and 4) siblings, if there is no one corresponding to 1)-3). As for the witnessing, the requirement for verification is such that two family members need to witness the patient expressing his or her preferences for LST. However, the family members are different in the case of surrogate decision making, for the decision must be made by agreement of all relevant members. Family members, in this case, include: 1) spouses, 2) lineal descendants and ascendants with one degree of kinship (i.e. parents and children,) 3) lineal descendants and ascendants with two degrees of kinship (i.e. grandparents, and grandchildren) 4) siblings, if there is no one corresponding to 1)-3). Thus, the Korean Act and the Taiwan Act differ, both in how they rank family members and how the family’s will is decided when there is no consensus.

In Japan, families have conventionally made medical decisions on behalf of patients [78, 79]. The draft AD Bill contains no provisions about the family’s ability to forgo treatment for patients. The Process Guideline, however, grants this role to the family of the patient, especially when the patient is incompetent and cannot express his or her will. In that case, the family either presumes the patient’s will or, when that is not possible, the healthcare team discusses with the family what will be in the patient’s best interest. Interestingly, the commentary of the Process Guideline explains that ‘family’ is not restricted to family members as defined by law, but can include people like trusted friends of the patient and those who have supported the patient at the end of their life.

In England, the MCA does not specify who may or may not be an attorney (although he or she must be an adult, i.e. of 18 years or older). Thus, unlike the three Asian countries mentioned above, legally speaking, family members are not automatically authorised to make decisions about treatment on behalf of the patient who has lost his or her decision-making capacity. The legal forms of LPA list people whom the patient trusts and know well as examples of an attorney, i.e., spouses, partners, adult children, or good friends [80]. Where the patient has not made an LPA, the relevant decision-maker is still required to consult those caring for the patient or interested in his or her welfare; no limit is placed on who, precisely, should be consulted.

The general rule is that, when a proxy makes decisions on behalf of the patient, it is crucial to represent the patient’s prior wishes or his or her best interests. Based on our analysis, we believe that the following issues regarding the role of the family need careful examination: 1) whether or not family members are the best proxies or advocates for incompetent patients [81]; and 2) whether or not there is a chance that family members may prioritise their own interests over those of the patient because they want to reduce financial or other types of burdens or even to benefit financially, for example through inheritance [7]. In addition, there are other issues such as the definition and scope of family [82].^25^^,^^26^ Compared with England, the three Asian countries traditionally place a greater emphasis on families and communities than on individuals. Thus, patients will sometimes prefer to have their family and physician decide the course of their treatments rather than doing so themselves; such family-based medical decision-making is said to be an expression of filial piety [83–87]. Further studies will be needed to determine how a suitable framework for proxy decision-making can be established in these Asian countries.

### Decision-making in the case of a patient without family

Lastly, our comparative analysis found that decision-making with, about or for an incompetent patient who lacks family or close friends may pose a severe problem. Recently, the significant increase in dementia patients has become a global issue; as of 2015, 46.8 million people worldwide are living with dementia, and nearly 10 million new cases emerge every year [88]. Dementia poses an enormous financial burden both on society and the individual, as well as giving rise to such issues as stigma and social isolation [89]. In the context of end-of-life decision-making, the following question is especially important: who should make, or contribute to, decisions, when patients with dementia who lack family or close friends are unable to make treatment decisions for themselves? In the three Asian countries highlighted here, no specific regulatory framework covers such cases. However, Japan’s Process Guideline recommends the establishment of a committee consisting of various experts to deal with such complicated cases.

In England and Wales, the MCA makes provision for those incapacitated adults who lack close family or friends, namely, the IMCA service. The purpose of the IMCA service is to represent and support particularly vulnerable people who have no family or friends and who lack the capacity to make important decisions about (providing, withholding, or withdrawing) serious medical treatments and changes in accommodation [52]. Clinicians must involve an IMCA when a decision about serious medical treatments is to be made in relation to such patients. An IMCA will interview or meet in private with the person who lacks mental capacity and obtain the views of professionals and anybody else who can provide information about the wishes, feelings, beliefs, or values of the person. They work to determine any alternative options, may examine relevant records, and then must write a report on their findings. The number of IMCA referrals regarding serious medical treatments (including withholding or withdrawing ANH) was 2132 between April 2013 and March 2014 [90], but has been increasing every year since 2007. Among these, patients with dementia accounted for 21% (446 referrals).

The IMCA service of England and Wales is unique among the four countries compared in this article [91]. We suggest that when lawmakers and policymakers make regulatory frameworks concerning end-of-life care, they should discuss not only ADs (including those encompassing attorneys) and proxy decision-making by the patient’s family, but also make provision for cases in which people who lack ADs, family, or close friends lose the capacity to make such decisions. The reason for this is that there will always be those “unbefriended patients” who cannot be helped by any of the standard legal mechanisms that protect and promote autonomy through the use of ADs, family, friends, or court-appointed guardians [92].^27^ In such cases, establishing a service such as England’s IMCA is one option, but the use of clinical ethics support services, such as hospital-based committees, may be another option, as suggested by Japan’s Process Guideline. As one study has shown, ethics consultation may be useful for resolving ethical dilemmas that involve the forgoing of LST [93].

Some limitations of the present study should be noted. Because our main focus was on laws and guidelines, our findings may not fully reflect the actual practice of healthcare professionals in each of the four countries. Thus, the actual practices in each country may be more similar to that in other countries, even though legal and other stipulations may suggest otherwise. Furthermore, we could not compare data surrounding withdrawal of LST among these four countries due to the lack of year-round data from each [94–97]^28^. Future studies should assess the actual practice(s) surrounding end-of-life care of healthcare professionals and psycho-spiritual care in end-of-life care in the four countries. Additionally, in a pandemic or disaster situation such as the current COVID-19 pandemic, ethically complex issues such as the allocation of scarce healthcare resources may adversely affect end-of-life care. Future research could usefully address such situations [98].

## Conclusions

This study compared regulatory frameworks governing forgoing LST in Japan, Korea, Taiwan, and England, and presented five important legal and philosophical points: 1) the importance of defining the terminal stage and associated criteria for clinical judgment; 2) the importance of discussions on withdrawing LST in PVS or MND patients who are not terminal; 3) the importance of (re)considering any moral and legal differences between withholding and withdrawing treatments; this is disputed particularly in Asian countries; 4) the importance of ascertaining the family’s role in end-of-life decision-making; and 5) the importance of devising ways to deal with incompetent patients who lack family or ADs. Focusing on these five points will be important for the establishment of better end-of-life regulatory frameworks for these four countries and others.

## Data Availability

All materials that support our findings of this study are publicly available.

## Abbreviations

ACP: Advanced care planning
AD: Advance directive
AIDS: Acquired immune deficiency syndrome
ANH: Artificial nutrition and hydration
BMA: British Medical Association
COPD: Chronic obstructive pulmonary disease
DNR: Do not resuscitate
GMC: General Medical Council
ICU: Intensive-Care Unit
IMCA: Independent Mental Capacity Advocate
KAMS: Korean Association of Medical Societies
LPA: Lasting Powers of Attorney
LST: Life-Sustaining Treatment
MCA: Mental Capacity Act
MCS: Minimally Conscious State
MHLW: Ministry of Health, Labour and Welfare
MHW: Ministry of Health and Welfare
MND: Motor Neurone Disease
POLST: Physician Order for Life-Sustaining Treatment
PRAA: Patient Right to Autonomy Act
PVS: Persistent vegetative state

## Footnotes

1 In the United States, beginning with California’s enactment of the Natural Death Act in 1976, all states had enacted AD-related laws by 2000.

2 England comprises different legal systems; our focus will be on English law i.e. essentially the law of England and Wales.

3 See: <https://www.bma.org.uk/> (accessed 25 April 2019).

4 The General Medical Council (GMC) in England is an independent organisation and statutory regulator for the medical profession in England. Its guidance applies to doctors working in all four UK countries (i.e., England, Wales, Scotland, and Northern Ireland). In addition, the GMC helps protect patients and improve medical education and practice.

5 See: <https://www.gmc-uk.org/> (accessed 25 April 2019).

6 On 28 April 2016, one of the authors (TANAKA) was able to view this important report which the hospital had submitted to the Shinminato District Police on 17 October 2005, by gaining access to the official files of Imizu City.

7 ALS is a rare neurological disease that mainly involves nerve cells (neurons) responsible for controlling voluntary muscle movements that enable actions such as chewing, walking, and talking. Currently, there is no cure for ALS and no effective treatment to halt, or reverse, the progression of the disease.

8 See: <https://www.ninds.nih.gov/Disorders/Patient-Caregiver-Education/Fact-Sheets/Amyotrophic-Lateral-Sclerosis-ALS-Fact-Sheet> (accessed 25 April 2019).

9 See: <http://www.nanbyou.or.jp/entry/52> (accessed 25 April 2019) (in Japanese).

10 Prior to this, a group of non-partisan Diet Members had announced a draft proposal in 2007 that would allow withdrawal of LST, but this was only an outline of a bill and was never submitted to the National Diet. The outline of the draft AD bill in 2012 was made public at the end of 2011. The initial plan allowed only terminally ill patients to withhold LST.

11 In the strict sense, the patient was near PVS (her brain responded to external stimuli, that she has minimal consciousness) but medical professionals expected she would not survive the condition very long. The Court recognised the professionals’ prediction, so the Court mentioned it as “irreversible dying stage”. So it ruled that the hospital should respect the patient’s preference. Ironically she regained the respiration ability, and lived another 7 months after the court decision.

12 Other countries include Thailand and Singapore. Thailand enacted the National Health Act, B.E. 2550 in 2007. Singapore enacted the Advance Medical Directive Act in 1997. Discussion of the situation in these countries is beyond the scope of this paper.

13 See: <http://www.ilo.org/dyn/natlex/natlex4.detail?p_lang=en&p_isn=82872&p_country=THA&p_count=441> (accessed 25 April 2019).

14 See: <https://sso.agc.gov.sg/Act/AMDA1996> (accessed 25 April 2019).

15 All of these countries have a national healthcare system covering all citizens. As for differences, the three Asian countries have adopted the social insurance scheme for healthcare, for which citizens of these countries must pay some healthcare costs at the time they receive care. Meanwhile, England’s national healthcare system is run by tax money, and people do not have to pay when they receive medical care.

16 In Japan, administrative guidelines with no legal basis have neither direct legal force in courts nor any direct effects on the rights and duties of citizens. The Process Guideline has no legal basis, and lack penal or administrative sanctions. However, the guideline is widely understood to express the official position of the MHLW concerning end-of-life care, and is therefore often cited in relevant literature as well as in the media.

17 Incidentally, in 2015, the MHLW revised the title of the Process Guideline to “the Process Guideline for the Medical Decision-making at the Last Stage of Life (jinsei no saisyu dankai),” because the term “end-of-life (or terminal) care (shumatuski iryo)” was not popular. However, as was the case with the original Process Guideline, the revised Process Guideline did not define the phrase “the Last Stage of Life”. The MHLW again revised the Process Guideline in March 2018, both to extend it to home-based care situations, and to include the idea of ACP.

18 The LST Decision Act uses the term “Advance Statement on Life-Sustaining Treatment” for conventional AD.

19 The GMC guidance also addresses decision-making for children, including neonates, and young people. Further details are provided in the following guidance.

20 The guidance defines the phrase as follows: for the purposes of this guidance, patients are ‘approaching the end of life’ when they are likely to die within the next 12 months. This includes patients for whom death is imminent (expected within a few hours or days) and those with: (a) advanced, progressive, incurable conditions; (b) general frailty and co-existing conditions that mean they are expected to die within 12 months; (c) existing conditions if they are at risk of dying from a sudden acute crisis in their condition; and (d) life-threatening acute conditions caused by sudden catastrophic events.

21 The revised law deleted these specific names of disease, and only provided the criteria of terminal stage. The revised law was enforced from 28 March, 2019.

22 In the commentary for the Process Guideline published by the MHLW, the terminal stage is not defined but the following situations are considered as terminal stage: 1) a patient’s prognosis is predicted to be from a few days to 2–3 months in the case of terminal cancer; 2) a patient repeats acute exacerbation of chronic disease and falls into poor prognosis; 3) a patient will die within months to years as the sequelae of cerebrovascular disease or senile deterioration. Likewise, the Science Council of Japan explains in the report that depending on the disease and condition of a patient, terminal stage can be divided into an acute type (e.g., emergency medical care), a subacute type (e.g., cancer), and a chronic type (e.g., senility, vegetative state, and dementia).

23 Actually, some state laws related to AD in the United States, including those in New Jersey and Tennessee, stipulate a definition of terminal stage.

24 In fact, the ALS patient who requested his mother to remove his artificial ventilator died after she removed it. The Yokohama District Court sentenced his mother for inducing or aiding suicide or homicide with consent in the case.

25 See (A lineal relative by affinity refers to a lineal relation of his or her spouse (such as a husband’s or wife’s parents or grandparents), and a spouse of his or her lineal relation (a husband or wife such as a child or a grandchild)) for different interpretations of family members in Korea’s and Taiwan’s Acts.

26 England’s MCA does not give such authority to family members both on decision-making and consent on behalf of individuals.

27 For example, there are more than 70,000 unbefriended patients and long-term care residents in the United States. In addition, two significant hospital studies found that 16% of patients admitted to an intensive care unit (ICU) and 5% of patients who died in the ICU were unbefriended.

28 While some comparative studies concerning forgoing LST have been conducted in European countries, to date there have been no such comparative international studies in Asian countries.
